# Evaluation of Cerebral Blood Flow and Cerebral Autoregulation Using Synthetic Data and In Silico Modeling

**DOI:** 10.1002/cns.70821

**Published:** 2026-03-12

**Authors:** Selim Bozkurt

**Affiliations:** ^1^ School of Engineering Ulster University Belfast UK

**Keywords:** cerebral autoregulation, cerebral blood flow, simulations, synthetic data

## Abstract

**Aims:**

The classic view of static cerebral autoregulatory function suggests that cerebral blood flow (CBF) remains relatively stable across a wide range of mean arterial pressure (MAP). Recent studies propose a narrow autoregulatory range, as the methods used to manipulate MAP may also influence CBF. This study evaluates static cerebral autoregulatory function using simulations and synthetic data, providing a theoretical framework for understanding static autoregulatory mechanisms in controlled conditions.

**Methods:**

Data for the relation between MAP and CBF were generated using a numerical model simulating the cardio and cerebrovascular systems and CBF regulation mechanisms. The influence of the cardio and cerebrovascular parameters on CBF was evaluated utilizing sensitivity analyses, and the independent effects of the hemodynamic parameters on CBF were assessed using partial regression analysis.

**Results:**

Sensitivity analysis showed that CBF is influenced by the systemic arteriolar resistance and arterial CO_2_ pressure. Partial regression analysis showed that the systemic arteriolar resistance and arterial CO_2_ pressure had significant effects on CBF, and the effect of MAP on CBF was significant, with a weak correlation.

**Conclusion:**

Synthetic data and simulations are feasible to evaluate static cerebral autoregulation and provide a theoretical framework for understanding mechanisms in controlled conditions.

## Graphical abstract

This study uses simulations and synthetic data to reassess static cerebral autoregulation. Results show cerebral blood flow is strongly influenced by systemic arteriolar resistance and arterial CO_2_, while mean arterial pressure has a significant but weak effect. Simulations offer a standardized framework for studying autoregulatory mechanisms under controlled conditions.

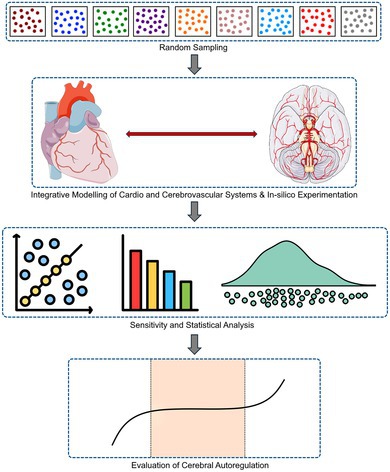

## 1 Introduction

Cerebral autoregulation is a physiological mechanism that maintains sufficient blood flow in the cerebral circulation despite the profound changes in the arterial blood pressure by controlling the cerebral arterial and arteriolar diameters [1]. The static cerebral autoregulatory function keeps the cerebral blood flow (CBF) relatively stable over a range of mean arterial pressure (MAP), whereas the dynamic cerebral autoregulatory function involves short‐term regulation of vascular tone to regulate the blood flow relatively quickly compared to the static cerebral autoregulation [2].

The classic view of the static cerebral autoregulatory function is that the CBF remains relatively stable over a wide range of MAP [3]. The classic view of the static cerebral autoregulatory mechanisms has been challenged in recent years, and a narrow autoregulation range has been proposed [4]. Differences across studies have been attributed to factors such as pharmacological manipulation of CBF and arterial CO_2_ pressure ($p_{{\mathrm{aCO}}_{2}}$) [5], the magnitude and timing of MAP changes [6], and the methodologies used to measure CBF [7]. The debate on the CBF autoregulation range is dynamic and ongoing, reflecting the complexity and evolving understanding of this physiological process. For instance, Classen [6] argued against the proposed static cerebral autoregulatory range in [7], stating that the classic static autoregulation describes CBF as a very stable process over a wide range of MAP, and the stability of CBF is profoundly influenced by the timing of the changes in MAP. Paulson et al. [8] argued that the data used in [7] were not normalized, and normalizing the data results in a wide range of static cerebral autoregulatory function.

As mentioned above, the selection of the experimental procedure, measurement method of CBF, the effect of the arterial CO_2_ pressure ($p_{{\mathrm{aCO}}_{2}}$) on CBF during the manipulation of MAP, or the effect of the dynamic cerebral autoregulation may complicate the measurement of CBF over a wide range of MAP [5, 6]. Moreover, evaluating cerebral blood velocity rather than directly measuring CBF is often considered more practical, as direct CBF measurement typically relies on techniques such as positron emission tomography or magnetic resonance imaging (MRI), which may involve specialized equipment and, in some cases, contrast agents [9].

The ongoing debate about the static cerebral autoregulation underscores the need for complementary and novel approaches to its investigation. Alternative methodologies, such as in silico experimentation, may provide additional insights into static cerebral autoregulatory mechanisms by enabling systematic control of physiological variables and isolation of specific regulatory components that are difficult to achieve in experimental or clinical settings. High fidelity computational models and the use of synthetic data offer opportunities to evaluate physiological systems by controlling factors which have profound effects on the physiological mechanisms [10, 11]. For instance, high‐fidelity synthetic patient data identical or very similar to real patients was generated using machine learning [12] or a sensitivity analysis was utilized to analyze the hemodynamic response of the model after hepatectomy and evaluate the surgical outcome [13]. A similar approach can also be used to evaluate the relationship between MAP and CBF while controlling or combining the effects of factors influencing these two parameters. The aim of this study is to evaluate static cerebral autoregulatory function using a computational model and synthetic data, providing a theoretical framework for understanding static autoregulatory mechanisms in controlled conditions.

## 2 Methods

**FIGURE 1:**
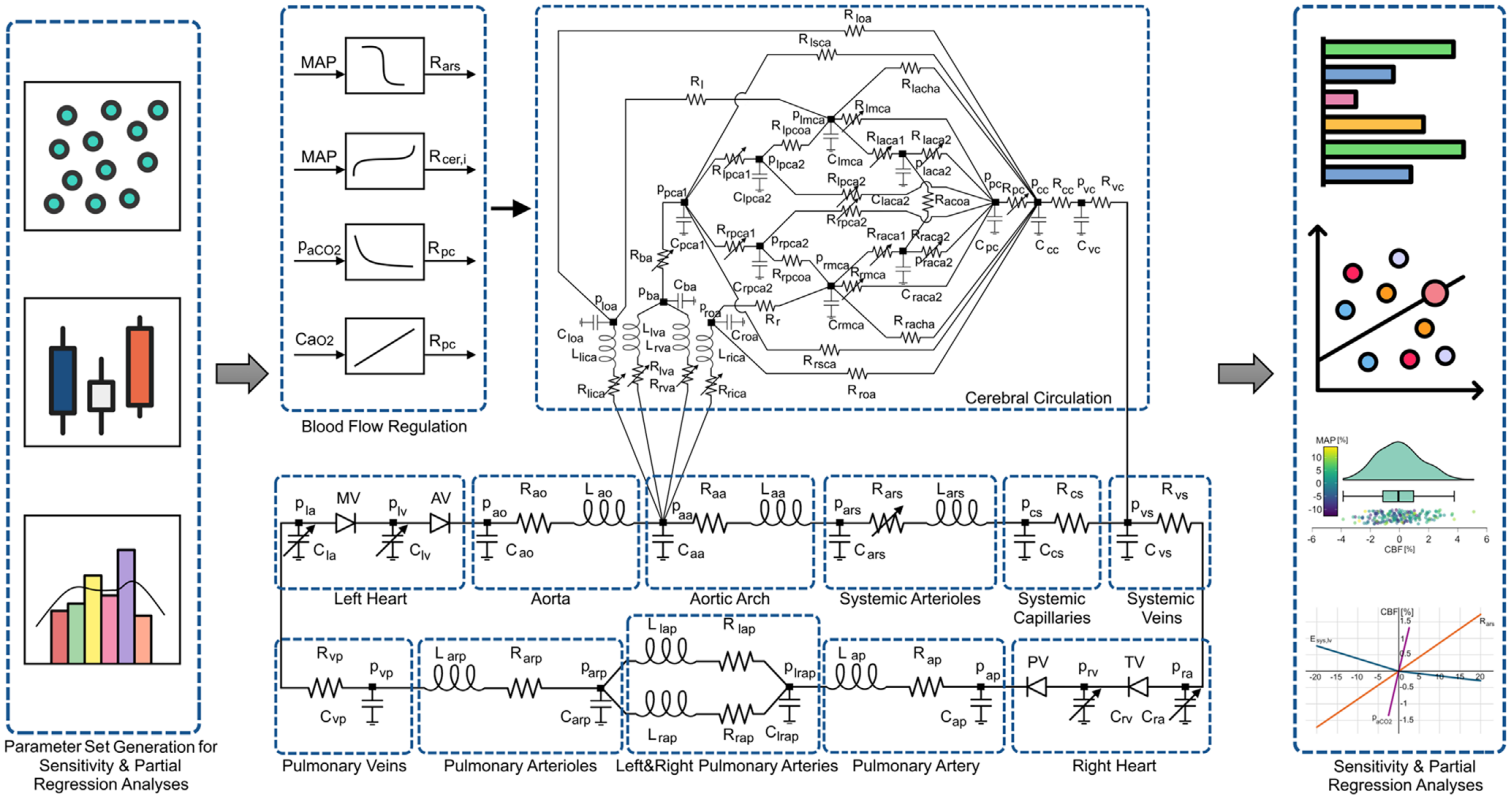
The workflow for evaluating cerebral autoregulation involves generating a parameter set to integrate into the cardio and cerebrovascular system model, followed by sensitivity analysis and partial regression analysis. Abbreviations used in the electrical analogue diagram of the cardio and cerebrovascular model are given in Table S1.

Data for the relation between MAP and CBF were generated using a numerical model simulating the cardio and cerebrovascular systems. The numerical model includes cardiac function, blood flow in systemic, pulmonary, and cerebral circulations, and CO_2_ and O_2_ contents in blood. Blood flow in the cerebral circulation is regulated through the interaction of the static cerebral autoregulatory function and cerebrovascular CO_2_ and O_2_ reactivities in the cerebrovascular system model. The influence of the cerebrovascular and cardiovascular parameters on the cerebral autoregulation was evaluated utilizing sensitivity analyses, and the independent effects of the hemodynamic parameters on the cerebral autoregulation were assessed using partial regression analysis. The workflow to evaluate the cerebral autoregulation and the electric‐analogue of the cardiovascular system model is given in Figure 1.

The pulsatile nature of the cardiovascular flow and pressures simulations in the systemic, pulmonary, and cerebral circulations was generated and regulated using coupled lumped‐parameter models implemented in MATLAB 2021b (Mathworks Inc., Natick, Massachusetts, USA). The cardiac and cerebrovascular systems are linked through shared pressures, flow rates, and regulatory mechanisms, including baroreflex control, cerebral autoregulation, and blood gas reactivity, as illustrated in the electric‐analogue diagram given in Figure 1.

### 2.1 Cardio and Cerebrovascular System Model

The cardiovascular system model simulates functions of the atria and ventricles, blood flow rate and pressure in the systemic and pulmonary circulations. Ventricular pressures (*p*_*v*_) in the cardiovascular system include active (*p*_*v,a*_) and passive (*p*_*v,p*_) components. The active pressure (*p*_*v,a*_) and volume (*V*_*v*_) relation in the left ventricle was modeled using end‐systolic elastance (*E*_*es,lv*_) and a time‐varying activation function (*f*_*act,v*_), whereas the left ventricular passive pressure and volume relation was described using an exponential function as follows:

(1) $$p_{\mathrm{lv}}=p_{\mathrm{lv},a}+p_{\mathrm{lv},p}$$

(2) $$p_{\mathrm{lv},a}\left(t\right)=E_{\mathrm{es},\mathrm{lv}}\left(V_{\mathrm{lv}}-V_{\mathrm{lv},0}\right)f_{\mathrm{act},\mathrm{lv}}\left(t\right)$$

(3) $$p_{\mathrm{lv},p}=A\left(e^{B\left(V_{\mathrm{lv}}-V_{\mathrm{lv},0}\right)}-1\right)$$

The left ventricular volume (*V*_*lv*_) was simulated using the radius at the base (*r*_*lv*_), longitudinal dimension (*l*_*lv*_), and an additional coefficient (*K*_*lv*_). The left ventricular inflow and outflow (*Q*_*mv*_ and *Q*_*av*_) were used to describe the change of the left ventricular radius at the base (dr_lv_/dt).

(4) $$V_{\mathrm{lv}}=\frac{2}{3}\pi K_{\mathrm{lv}}r_{\mathrm{lv}}^{2}l_{\mathrm{lv}}$$

(5) $$\frac{{\mathrm{dr}}_{\mathrm{lv}}}{\mathrm{dt}}=\frac{3\left(Q_{\mathrm{mv}}-Q_{\mathrm{av}}\right)}{4\pi K_{\mathrm{lv}}l_{\mathrm{lv}}}{\left(\frac{3V_{\mathrm{lv}}}{2\pi K_{\mathrm{lv}}l_{\mathrm{lv}}}\right)}^{-1/2}$$

Right ventricular blood pressure (*p*_*la*_) and volume (*V*_*la*_) were described similarly, using different parameter values. Detailed information about the modeling of the ventricular functions can be found in [14]. Left atrial pressure and volume relation was simulated using a time‐varying left atrial elastance function (*E*_*la*_) as given below:

(6) $$p_{\mathrm{la}}\left(t\right)=E_{\mathrm{la}}\left(t\right)\left(V_{\mathrm{la}}-V_{\mathrm{la},0}\right)$$

Left atrial volume was described using the left atrial radius (*r*_*la*_), longitudinal dimension (l_la_) and a scaling coefficient (*K*_*la*_). Left atrial inflow and outflow (*Q*_pv_ and *Q*_*mv*_), as in the left ventricle, were used to describe the change of the left atrial radius over time (dr_la_/dt).

(7) $$V_{\mathrm{la}}=\frac{2}{3}\pi K_{\mathrm{la}}r_{\mathrm{la}}^{2}l_{\mathrm{la}}$$

(8) $$\frac{{\mathrm{dr}}_{\mathrm{la}}}{\mathrm{dt}}=\frac{3\left(Q_{\mathrm{pv}}-Q_{\mathrm{mv}}\right)}{4\pi K_{\mathrm{la}}l_{\mathrm{la}}}{\left(\frac{3V_{\mathrm{la}}}{2\pi K_{\mathrm{la}}l_{\mathrm{la}}}\right)}^{-1/2}$$

Right atrial function was simulated similarly using different parameter values. Detailed information about the simulation of the atrial functions can be found in [14]. Blood pressures in the systemic and pulmonary circulations were modeled using equivalent electrical analogue models as described in [15].

Blood flow in the vascular compartments was modeled considering resistance (R), compliance (C), and inertance (L) properties of the blood vessels. Change of the pressure and flow rate over time (dP/dt and dQ/dt) in the vascular compartments was simulated as follows:

(9) $$\frac{dp_{i}}{\mathrm{dt}}=\frac{Q_{i-1}-Q_{i}}{C_{i}}$$

(10) $$\frac{dQ_{i}}{\mathrm{dt}}=\frac{p_{i}-p_{i+1}-Q_{i}R_{i}}{L_{i}}$$

Here, *p* and *Q* represent pressure and flow rate, and subscript i represents the vascular compartments that are modeled in the cardio and cerebrovascular system model.

Systemic blood flow rate was regulated by a baroreflex model, which controls the systemic arteriolar resistance (*R*_*ars*_). The baroreflex model includes the mean aortic pressure (*p*_*ao,m*_), a sensitivity coefficient for the systemic arteriolar resistance (*S*_*Rars*_) and setpoints for aortic pressure and systemic arteriolar resistance (*p*_*ao,ars,set*_, *R*_*ars,set*_) as described in [16, 17].

(11) $$\Delta R_{\mathrm{ars}}=\left|S_{\text{Rars}}\left(p_{\mathrm{ao},\mathrm{ars},\mathrm{set}}-p_{\mathrm{ao},m}\right)R_{\mathrm{ars},\mathrm{set}}\right|$$

(12) $$R_{\mathrm{ars}}=\left\{\begin{array}{l} R_{\mathrm{ars},\mathrm{set}}-\Delta R_{\mathrm{ars}}\;p_{\mathrm{ao},m}\geq p_{\mathrm{ao},\mathrm{ars},\mathrm{set}}\\ R_{\mathrm{ars},\mathrm{set}}+\Delta R_{\mathrm{ars}}\;p_{\mathrm{ao},m}<p_{\mathrm{ao},\mathrm{ars},\mathrm{set}} \end{array}\right.$$

Cerebrovascular system model simulates blood flow rates and pressures in the internal carotid and vertebral arteries, basilar artery, anterior, middle, and posterior cerebral arteries, anterior choroidal arteries, superior cerebellar arteries, ophthalmic arteries, anterior and posterior communicating arteries, pial circulation, and cerebral capillaries and veins.

Blood flow in the cerebral circulation is regulated by modeling the interaction of the static cerebral autoregulation, cerebrovascular CO_2_ and O_2_ reactivities. Previous studies have shown that cerebral autoregulation can be assessed at middle at the internal carotid arteries [18], basilar and vertebral arteries [19], and the middle cerebral arteries [20]. Blood flow is also regulated in the anterior and posterior cerebral arteries to varying degrees [21]. Moreover, pial circulation is one of the main compartments of cerebral blood flow regulation with respect to mean arterial blood pressure changes [22, 23]. Therefore, a static autoregulatory function controlling the resistances of the above‐mentioned arteries and pial arterioles was adapted from [16] to regulate blood flow rate in the cerebral circulation.

(13) $$\Delta R_{i,\mathrm{pao}}=\left|S_{i}\left(p_{\mathrm{ao},i,\mathrm{set}}-p_{\mathrm{ao},m}\right)R_{i,\mathrm{set}}\right|$$

(14) $$R_{i,\mathrm{pao}}=\left\{\begin{array}{l} R_{i,\mathrm{set}}+\Delta R_{i,\mathrm{pao}}\;p_{\mathrm{ao},m}>p_{\mathrm{ao},i,\mathrm{set}}\\ R_{i,\mathrm{set}}-\Delta R_{i,\mathrm{pao}}\;p_{\mathrm{ao},m}\leq p_{\mathrm{ao},i,\mathrm{set}} \end{array}\right.$$

Here, Δ*R*_*i,ao*_ represents the change in the arterial or arteriolar resistance, S_i_ represents the sensitivity of the arterial or arteriolar compartment to changes in the mean arterial pressure, p_ao,i,set_ and p_ao,m_ represent the arterial pressure set points and mean aortic pressure, and R_i,set_ represents the set point for the arterial or arteriolar resistance. The set values for resistance, inertance, and compliance in the cerebrovascular compartments are given in Table S2.

Cerebrovascular CO_2_ and O_2_ reactivities regulate the blood flow rate in the cerebral arteries by controlling pial arteriolar resistance, as given below:

(15) $$R_{\mathrm{pc},CO_{2}}=R_{\mathrm{pc},\mathrm{set}}*a_{1}*e^{a_{2}*p_{a,{\mathrm{CO}}_{2}}}$$

(16) $$R_{\mathrm{pc},O_{2}}=R_{\mathrm{pc},\mathrm{set}}\left(c_{1}\times {C_{a,O}}_{2}+c_{2}\right)$$

Here, $R_{\mathrm{pc},{\mathrm{CO}}_{2}}$ and $R_{\mathrm{pc},O_{2}}$ represent pial arteriolar resistance controlled by the arterial CO_2_ pressure (${p_{a,\mathrm{CO}}}_{2}$) and O_2_ content (${C_{a,O}}_{2}$), whereas a_1_, a_2_, c_1_, and c_2_ are the coefficients. Detailed information about the modeling of the blood flow rate regulation by the arterial CO_2_ pressure (${p_{a,\mathrm{CO}}}_{2}$) and O_2_ content (${C_{a,O}}_{2}$) can be found in [24, 25].

O_2_ contents (${C_{a,O}}_{2}$) in the arterial blood were described using O_2_ binding capacity, saturation (${S_{a,O}}_{2}$) and dissolved O_2_ in plasma [26, 27]. Arterial CO_2_ pressure (${p_{a,\mathrm{CO}}}_{2}$) was modeled using arterial CO_2_ concentration ($C_{a,{\mathrm{CO}}_{2},p}$) in the plasma, reference blood pH and arterial blood pH in each compartment (pH_a_) and hemoglobin level in the blood (Hb) [28].

(17) $${C_{a,O}}_{2}=1.34\mathrm{Hb}\times {S_{a,O}}_{2}+0.0031p_{a,O_{2}}$$

(18) $$p_{a,{\mathrm{CO}}_{2}}=\frac{C_{a,CO_{2},p}-\left(\mathrm{pH}-{\mathrm{pH}}_{a}\right)\left(-18.2532-3.103044\mathrm{Hb}\right)}{0.06868\times 10^{1.0424\mathrm{pHa}-6.410361}}$$

Detailed information about the incorporation of the arterial O_2_ content and CO_2_ blood pressure can be found in [25].

### 2.2 Sensitivity and Partial Regression Analyses

Static cerebral autoregulation is evaluated by manipulating the mean arterial pressure using different methods, such as pharmacological methods, including drugs like phenylephrine to increase systemic arteriolar resistance and arterial blood pressure [2], noninvasive methods in which the effect of dynamic cerebral autoregulation on the cerebral blood flow diminishes [7, 29], or head up or down tilt tests [30, 31]. CO_2_ correction may be required in tests in which arterial CO_2_ pressure (${p_{a,\mathrm{CO}}}_{2}$) is uncontrolled [7].

Influence of the systemic arteriolar resistance (R_ars_) left ventricular systolic elastance (E_sys,lv_), arterial CO_2_ pressure (${p_{a,\mathrm{CO}}}_{2}$), and sensitivities (S_i_) of the internal carotid and vertebral arteries, basilar artery, anterior, middle and posterior cerebral arteries, and pial arterioles on the mean arterial pressure and cerebral blood flow utilizing sensitivity analysis taking into account direct relationships between the input and out variables. Partial regression analysis was used to evaluate the controlled effects of change in systemic arteriolar resistance (R_ars_) left ventricular systolic elastance (E_sys,lv_), and arterial CO_2_ pressure (${p_{a,\mathrm{CO}}}_{2}$) on the change in the cerebral blood flow rate. Also, the correlation between the change in the mean arterial pressure and cerebral blood flow was evaluated using partial regression analysis. The systemic arteriolar resistance (R_ars_) left ventricular systolic elastance (E_sys,lv_), and arterial CO_2_ pressure (${p_{a,\mathrm{CO}}}_{2}$) were selected to include in the sensitivity and partial regression analyses, considering the mean arterial pressure manipulation methods mentioned above. The sensitivities (S_i_) of the cerebral arteries and arterioles were included in the sensitivity analyses because this parameter controls the cerebral arterial and arteriolar resistances in the cerebrovascular model.

**FIGURE 2:**
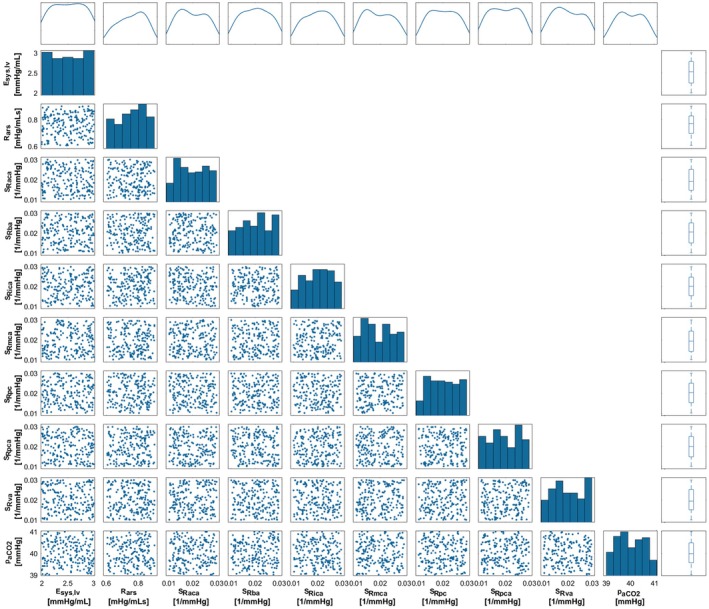
The scatter plots, histograms, density plots, and boxplots for the sampled parameters in the sensitivity analysis. E, R, S, and p represent elastance, resistance, sensitivity and pressure, subscripts sys, lv, ars, R, aca, ba, ica, mca, pc, pca, va, and aCO_2_ represent systolic, left ventricle, systemic arterioles, resistance, anterior cerebral artery, internal carotid artery, middle cerebral artery, pial circulation, posterior cerebral artery, vertebral artery, and arterial carbon dioxide.

Two hundred samples for each variable were generated in MATLAB 2021b (Mathworks Inc., Natick, Massachusetts, USA), and the Simulink Sensitivity Analyzer in MATLAB 2021b (Mathworks Inc., Natick, Massachusetts, USA) was used to incorporate parameter values into the cardio and cerebrovascular model. The relationship between the cerebral blood flow and mean arterial pressure for the generated data was evaluated by fitting a linear curve using the Curve Fitting Tool in MATLAB 2021b (Mathworks Inc., Natick, Massachusetts, USA). JASP 0.19.3.0 (Amsterdam, Netherlands) was used to perform partial regression analyses. The scatter plots, histograms, density plots, and boxplots for the sampled parameters in the sensitivity analysis are given in Figure 2. The set values, lower and upper limits, mean values and standard deviations for the sampled parameters are given in Table 1.

**TABLE 1:** The set values, lower and upper limits, mean values, and standard deviations for the sampled parameters. E, R, S, and p represent elastance, resistance, sensitivity, and pressure, while subscripts sys, lv, R, ars, aca, ba, ica, mca, pc, pca, va, and aCO_2_ represent systolic, left ventricle, systemic arterioles, resistance, anterior cerebral artery, internal carotid artery, middle cerebral artery, pial circulation, posterior cerebral artery, vertebral artery, and arterial carbon dioxide.

	Set value	Lower limit	Upper limit	Mean ± SD
E_sys,lv_ [mmHg/mL]	2.5	2	3	2.5 ± 0.31
R_ars_ [mmHg/mLs]	0.75	0.6	0.9	0.76 ± 0.08
S_Rica_ [1/mmHg]	0.02	0.01	0.03	0.020 ± 0.005
S_Rva_ [1/mmHg]	0.02	0.01	0.03	0.020 ± 0.006
S_Rba_ [1/mmHg]	0.02	0.01	0.03	0.020 ± 0.006
S_Raca_ [1/mmHg]	0.02	0.01	0.03	0.020 ± 0.006
S_Rmca_ [1/mmHg]	0.02	0.01	0.03	0.019 ± 0.006
S_Rpca_ [1/mmHg]	0.02	0.01	0.03	0.020 ± 0.006
S_Rpca_ [1/mmHg]	0.02	0.01	0.03	0.020 ± 0.006
$p_{{\mathrm{aCO}}_{2}}$ [mmHg]	40	39	41	40 ± 0.54

## 3 Results

Initially, a simulation was performed to evaluate the cardiac function and the cerebral blood flow in the cardio and cerebrovascular system model at set values for the evaluated variables in the sensitivity analysis. Cardiac, aortic, and pulmonary arterial pressures, cardiac volumes are given in Figure S1, and the cerebrovascular blood flow rates are given in Figure S2.

**FIGURE 3:**
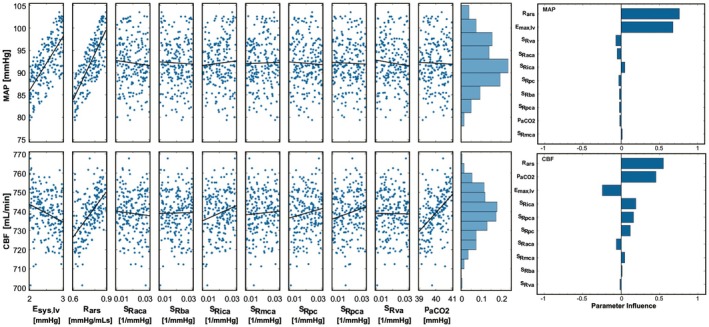
Scatter plots with linear overlay fit and the tornado plots presenting the influence of the left ventricular systolic elastance, systemic arteriolar resistance, arterial CO_2_ pressure and the cerebral arterial and arteriolar sensitivities on the mean arterial pressure and cerebral blood flow. E, R, S, and p represent elastance, resistance, sensitivity, and pressure; subscripts, sys, lv, R, ars, aca, ba, ica, mca, pc, pca, va, and aCO_2_ represent systolic, left ventricle, systemic arterioles, resistance, anterior cerebral artery, internal carotid artery, middle cerebral artery, pial circulation, posterior cerebral artery, vertebral artery, and arterial carbon dioxide.

Scatter plots with linear overlay fit and the tornado plots presenting the influence of the left ventricular systolic elastance (E_sys,lv_), systemic arteriolar resistance (R_ars_), arterial CO_2_ pressure ($p_{{\mathrm{aCO}}_{2}}$), and the cerebral arterial and arteriolar sensitivities (S_i_) on the mean arterial pressure and cerebral blood flow rate are given in Figure 3.

The systemic arteriolar resistance (R_ars_) and left ventricular systolic elastance (E_sys,lv_) highly influenced the mean arterial pressure, whereas the arterial CO_2_ pressure ($p_{{\mathrm{aCO}}_{2}}$) and the cerebral arterial and arteriolar sensitivities (S_i_) did not have a profound effect on the mean arterial pressure. Correlation coefficients for the influence of the systemic arteriolar resistance (R_ars_) and left ventricular systolic elastance (E_sys,lv_) on the mean arterial pressure were 0.76 and 0.68, respectively. The correlation coefficients for the arterial CO_2_ pressure ($p_{{\mathrm{aCO}}_{2}}$) and the cerebral arterial and arteriolar sensitivities (S_i_) remained between 0.1 and −0.1. Ranges of the MAP and CBF were between 79.4 mmHg and 103.5 mmHg and 701 mL/min and 768 mL/min, with mean values of 92.1 ± 5.4 mmHg and 739 ± 11.4 mL/min, respectively.

The systemic arteriolar resistance (R_ars_) and arterial CO_2_ pressure ($p_{{\mathrm{aCO}}_{2}}$) profoundly affected the cerebral blood flow rate. Correlation coefficients for the influence of the systemic arteriolar resistance (R_ars_) and arterial CO_2_ pressure ($p_{{\mathrm{aCO}}_{2}}$) on the cerebral blood flow rate were 0.55 and 0.46, respectively. Also, the coefficient of correlation for the influence of the left ventricular systolic elastance on the cerebral blood flow rate was −0.24. The correlation coefficients for the cerebral arterial and arteriolar sensitivities (S_i_) remained between 0.2 and −0.2.

**FIGURE 4:**
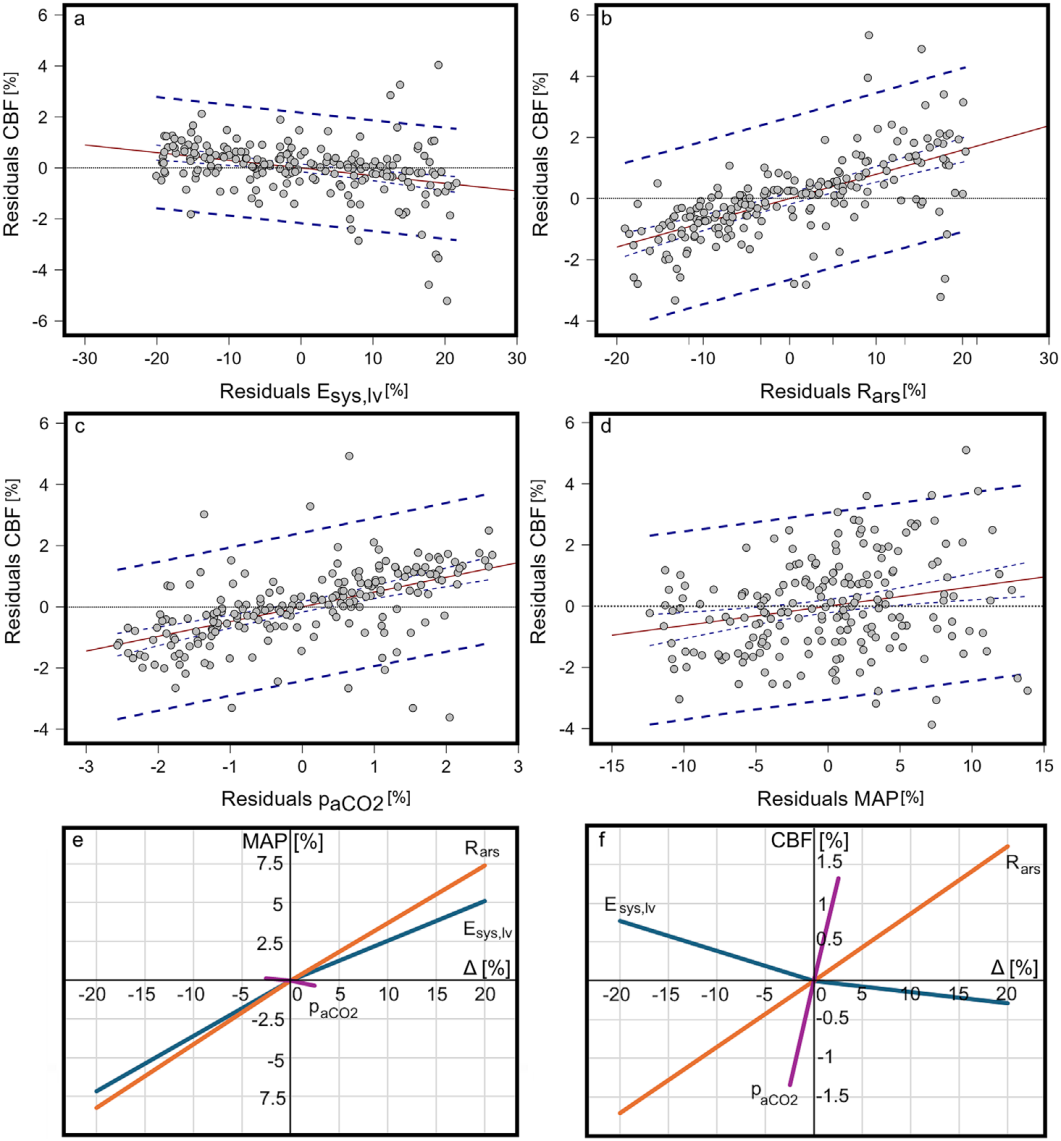
Partial regression plot showing the controlled effects of the changes in the (a) left ventricular systolic elastance (E_sys,lv_), (b) systemic arteriolar resistance (R_ars_), (c) arterial CO_2_ pressure ($p_{{\mathrm{aCO}}_{2}}$), (d) mean arterial pressure (MAP) on the cerebral blood flow (CBF), and effects of the maximal increase and decrease within the selected ranges of the left ventricular systolic elastance (E_sys,lv_), systemic arteriolar resistance (R_ars_) and the arterial CO_2_ pressure ($p_{{\mathrm{aCO}}_{2}}$) on the (e) mean arterial pressure (MAP) and (f) cerebral blood flow rate (CBF).

Partial regression plots showing the controlled effects of the changes in the left ventricular systolic elastance (E_sys,lv_), systemic arteriolar resistance (R_ars_), and the arterial CO_2_ pressure ($p_{{\mathrm{aCO}}_{2}}$) on the change in the cerebral blood flow rate and correlation between the change in the mean arterial pressure and the change in the cerebral blood flow rate, as well as the effects of the maximal increase and decrease within the selected ranges of the left ventricular systolic elastance (E_sys,lv_), systemic arteriolar resistance (R_ars_), and the arterial CO_2_ pressure ($p_{{\mathrm{aCO}}_{2}}$) on the mean arterial pressure and cerebral blood flow rate are given in Figure 4.

**FIGURE 5:**
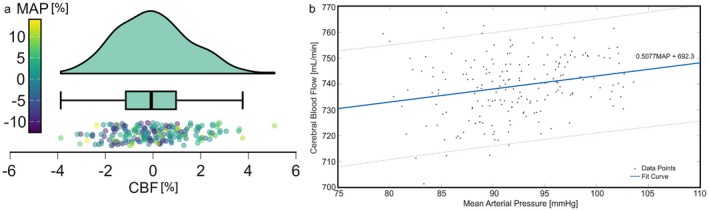
(a) Raincloud plot, probability density, and boxplots for the distribution of the changes in the mean arterial pressure (MAP) and cerebral blood flow (CBF), (b) linear fit curve showing the relationship between the cerebral blood flow and mean arterial pressure for the generated data with 95% prediction interval.

Changes in the left ventricular systolic elastance (E_sys,lv_), systemic arteriolar resistance (R_ars_), and the arterial CO_2_ pressure ($p_{{\mathrm{aCO}}_{2}}$) affected the change in the CBF significantly (Figure 4a–c). The partial correlation coefficients between the CBF and left ventricular systolic elastance (E_sys,lv_), systemic arteriolar resistance (R_ars_), and the arterial CO_2_ pressure ($p_{{\mathrm{aCO}}_{2}}$) were −0.34, 0.64, and 0.54, respectively. Also, the change in the MAP had a significant effect on the change in the CBF (Figure 4d). However, the correlation coefficient for the effect of the change in the MAP on the change in the CBF was 0.24, showing a weak partial correlation between these two parameters. Increasing the left ventricular systolic elastance (E_sys,lv_) from the set point had a relatively small influence on the change in the mean arterial pressure and cerebral blood flow compared to decreasing it (Figure 4e,f). Increasing or decreasing the systemic arteriolar resistance (R_ars_) did not change the effect of this parameter on the MAP and CBF. Raincloud plot, probability density, and boxplots for the distribution of the changes in the MAP and CBF and linear fit curve showing the relationship between the cerebral blood flow and mean arterial pressure for the generated data with a 95% prediction interval are given in Figure 5.

The range of the change in the CBF was between −4% and 4%, except for one data point. However, the change in the MAP was approximately between −12% and 12%. The density of the data points shows that there is a higher likelihood of changes in the CBF between 1% and 1%. The slope of the fitted linear curve was 0.5077 mL/min/mmHg. Also, the curve fitting showed that increasing the MAP increases the CBF for the generated data.

## 4 Discussion

In this study, the effects of cardio and cerebrovascular parameters on MAP and CBF were evaluated by generating synthetic data and in silico experimentation. The relationship between CBF and MAP within cerebral autoregulation has been previously described [3]. Nevertheless, as mentioned, cerebral autoregulatory function is currently a topic of debate [4]. The purpose of this study is to introduce a novel in silico experimentation approach for analyzing cerebral autoregulation rather than examining established physiological relationships. Therefore, previously published literature was used to validate the proposed methodology.

The utilized static cerebral autoregulatory function was adopted from Bozkurt [16]. Bozkurt [16] modeled the static cerebral autoregulatory function to control the vascular resistance in the pial circulation. However, as mentioned before, blood flow in the cerebral arteries is also regulated in the major cerebral arteries as well [18, 19, 20, 21]. Therefore, the static cerebral autoregulatory function was extended to regulate the blood flow in the internal carotid, basilar, vertebral, middle, and posterior cerebral arteries, along with pial arterioles. The utilized cardio and cerebrovascular model incorporates vascular resistance as a parameter regulating the blood flow rate in the cerebral arteries. The static cerebral autoregulatory function, which controls the cerebral arterial and arteriolar resistances, utilizes arterial and arteriolar sensitivity, arterial pressure set points, and mean aortic pressure. Consequently, changes in arterial pressure, resistance set points, or vascular sensitivity directly influence the simulated cerebral blood flow rate. The utilized static cerebral autoregulatory function model simulates the interaction between the CBF and MAP (Equations 13 and 14).

The microvascular compartments were not modeled in the cerebrovascular system model. Small cerebral vessels and capillaries also contribute to the regulation of cerebral blood flow [32, 33, 34]. However, the utilized model focuses on pressure‐driven autoregulatory mechanisms in the major cerebral arteries and pial arterioles, in which the cerebral blood flow is regulated in response to changes in mean arterial pressure [35]. Incorporating microvascular dynamics would substantially increase model complexity and parameter uncertainty.

The scatter plots in Figure 2 show the distributions of the independently generated variables which were used to construct the virtual populations. Scatter plots provide an initial assessment of potential correlations between generated data. The absence of strong patterns in the scatter plots suggests that the variables are largely independent [36], which was with the intended data‐generation process. Histograms and density plots further demonstrate that the data are spread fairly even across their respective ranges and support the assumption that all values between the minimum and maximum were assigned fairly equal probabilities. Box plots summarize the spread of the generated data and show there was no unintended clustering or skewness in the dataset.

The effects of the systemic arteriolar resistance (R_ars_) and left ventricular systolic elastance (E_sys,lv_) were evaluated because pharmacological methods, such as the use of phenylephrine to manipulate MAP, alter the systemic arteriolar resistance (R_ars_) without affecting myocardial contractility [37]; however, methods like the head‐up tilt test may influence systemic resistance and ventricular contractility in the cardiovascular system [38]. Moreover, arterial CO_2_ pressure ($p_{{\mathrm{aCO}}_{2}}$) may remain uncontrolled during tests, altering the slope of the cerebral autoregulation curve [7]. The ranges of the systemic arteriolar resistance (R_ars_) and left ventricular systolic elastance (E_sys,lv_) in the generated data were selected to obtain regulated CBF values in the range of both classic and new autoregulatory curves [39]. The range of the arterial CO_2_ pressure ($p_{{\mathrm{aCO}}_{2}}$) was kept narrow, considering the data in the different experiments given in [7]. The range of the cerebral arterial and arterial sensitivities (S_i_) was defined iteratively to ensure a physiological response from the cerebrovascular model.

Sensitivity analysis showed that MAP is profoundly influenced by the systemic arteriolar resistance (R_ars_) and left ventricular systolic elastance (E_sys,lv_) whereas the systemic arteriolar resistance (R_ars_), arterial CO_2_ pressure ($p_{{\mathrm{aCO}}_{2}}$), and left ventricular systolic elastance (E_sys,lv_) are the most influential parameters on CBF. Here, it should be noted that the results are valid for the selected ranges and included parameters in the sensitivity analysis. It has already been shown that arterial CO_2_ pressure ($p_{{\mathrm{aCO}}_{2}}$) is more influential than the systemic arteriolar resistance (R_ars_) and left ventricular systolic elastance (E_sys,lv_) on CBF within a wider range [24]. Nonetheless, arterial CO_2_ pressure ($p_{{\mathrm{aCO}}_{2}}$) had a profound effect on CBF even within a narrow range in the simulations; therefore, CO_2_ levels must be manipulated in the tests evaluating cerebral autoregulation to ensure stable CBF despite changes in MAP. Left ventricular systolic elastance (E_sys,lv_) was negatively correlated with CBF; therefore, increasing myocardial contractility reduces CBF. Experimental studies show that increasing cardiac contractility affects internal carotid arterial blood flow negatively [40], confirming the results from the sensitivity analysis. Cerebral arterial and arteriolar sensitivities (S_i_) had slight effects on CBF. This may be because blood in the cerebral circulation is carried by multiple blood vessels, and the change in resistance of multiple compartments may have a slight effect on the overall CBF, as the results in the sensitivity analysis show.

Partial regression analysis is used to isolate the effect of one independent variable on the dependent variable while controlling for other variables. Partial correlation between the systemic arteriolar resistance (R_ars_), arterial CO_2_ pressure ($p_{{\mathrm{aCO}}_{2}}$), and left ventricular systolic elastance (E_sys,lv_) and the CBF were higher than the correlation coefficients. Such a result may indicate that the effects of the hemodynamic parameter on the CBF may attenuate each other. The effect of MAP was also significant; however, there was a weak positive correlation between MAP and CBF. Such a result shows that a substantial relative change in MAP results in smaller relative changes in CBF. Probability density distribution and box plot presented in Figure 5a confirm this.

The slope of the regression curve in Figure 5b was around 0.5 mL/min/mmHg. Experimental studies show that the change in the blood velocity in the middle cerebral artery is correlated with MAP, and the slope of the regression curve was 0.55 cm/s/mmHg [41].

MAP and mean pulmonary arterial pressure were 92.1 mmHg and 13.7 mmHg, cardiac output was 5.2 L/min and CBF was 732 mL/min (Table S3). These results were within a physiological range for healthy adults [26, 42]. Also, the average blood flow rates in the internal carotid and vertebral arteries, basilar artery and anterior, middle, and posterior cerebral arteries were around 275 mL/min, 92 mL/min, 184 mL/min, 76 mL/min, 119 mL/min, and 45 mL/min, respectively. Again, these results were within a physiological range [42, 43, 44] confirming the accuracy of the simulations and the capability of the numerical model utilized to simulate cardio and cerebrovascular systems.

Experimental or clinical datasets may be constrained by limited pressure ranges, interindividual variability, and ethical restrictions [45, 46], whereas synthetic data and in silico modeling allow independent manipulation of hemodynamic variables that influence vascular blood flow and pressure [47]. Therefore, the effects of the investigated parameters on cerebral blood flow can be evaluated under well‐controlled conditions, allowing direct interpretation of their contribution to static cerebral autoregulatory function. Moreover, in silico methods have the potential to transform into powerful translational tools such as Digital Twins and may improve cerebrovascular risk stratification and therapeutic evaluation in future [48]. For instance, impairment of static cerebral autoregulation is associated with chronic hypo or hyperperfusion and may contribute to the development of cerebrovascular diseases such as ischemic stroke and cognitive impairment [49]. Therefore, evaluation of static cerebral autoregulation may provide insights into risks associated with cerebrovascular events, and in silico methods may serve as a tool for exploring physiological and clinical scenarios relevant to cerebrovascular conditions.

Cerebral autoregulation in the cardio and cerebrovascular system model represents normal physiological control of cerebral blood flow through static autoregulation and cerebrovascular gas reactivity. CBF is maintained by controlling cerebral arterial and arteriolar resistances in response to changes in MAP, whereas cerebrovascular CO_2_ reactivity provides additional control on the CBF in the utilized numerical model. It should be acknowledged that computational models approximate biological mechanisms [50]. Physiological regulation of CBF involves complex, time‐dependent interactions among endothelial, metabolic, and neurogenic mechanisms that are simplified in computational models [1]. These results presented in this study can be interpreted as representations of average, ideal autoregulatory behavior rather than a complete depiction of all adaptive processes present in a healthy cerebrovascular system.

## 5 Conclusion

This study demonstrated the feasibility of the use of synthetic data to evaluate the static cerebral autoregulation curve. Sensitivity and partial regression analyses and curve fitting confirm the correlations between the cardio and cerebrovascular variables and MAP and CBF. Also, numerical simulations provide accurate results for hemodynamic variables in the cardiovascular system and blood flow rates in the cerebrovascular system. Therefore, the numerical model used in this study, in combination with synthetic data, can be used to evaluate physiological scenarios in future.

## Funding

The author has nothing to report.

## Ethics Statement

This study does not involve humans or animals; therefore, it does not require approval from an institutional ethics committee.

## Conflicts of Interest

The author declares no conflicts of interest.

## Supporting information


**Table S1:** Glossary of abbreviations.
**Table S2:** Set values for the resistance (R), inertance (L), and compliance (C) in the cerebrovascular compartments, left and right compartments assumed to have the same R, L, and C properties, therefore, the same parameter values were used in the simulations.
**Table S3:** Aortic pressure, cardiac output, total cerebral blood flow and blood flow rates through internal carotid arteries (ICA), vertebral arteries (VA), basilar artery (BA), anterior cerebral arteries (ACA), middle cerebral arteries (MCA), and posterior cerebral arteries (PCA) over a cardiac cycle at set values for the evaluated variables in the sensitivity analysis.
**Figure S1:** (a) Left ventricular and aortic pressure (p_lv_ and _pao_), (b) right ventricular and pulmonary arterial pressure (p_rv_ and p_ap_), and (c) left and right ventricular volume (V_lv_ and V_rv_) signal waveforms over a cardiac cycle.
**Figure S2:** (a) Internal carotid, vertebral and basilar arterial flow rate (Q_ica_, Q_va_, and Q_ba_) signal waveforms over a cardiac cycle, (b) anterior, middle, and posterior cerebral arterial flow rate (Q_aca_, Q_mca_, and Q_pca_) signal waveforms over a cardiac cycle.

## Data Availability

The data that support the findings of this study are available from the corresponding author upon reasonable request.
