# Supplementary material for: Evaluation of Cerebral Blood Flow and Cerebral Autoregulation Using Synthetic Data and In Silico Modeling
Source: CNS Neurosci Ther. 2026 Mar 12;32(3):e70821. doi: 10.1002/cns.70821 (PMC13093671; doi:10.1002/cns.70821)
Supplement: Supplementary file 1 — Table S1: Glossary of abbreviations. Table S2: Set values for the resistance (R), inertance (L), and compliance (C) in the cerebrovascular compartments, left and right compartments assumed to have the same R, L, and C properties, therefore, the same parameter values were used in the simulations. Table S3: Aortic pressure, cardiac output, total cerebral blood flow and blood flow rates through internal carotid arteries (ICA), vertebral arteries (VA), basilar artery (BA), anterior cerebral arteries (ACA), middle cerebral arteries (MCA), and posterior cerebral arteries (PCA) over a cardiac cycle at set values for the evaluated variables in the sensitivity analysis. Figure S1: (a) Left ventricular and aortic pressure (plv and pao), (b) right ventricular and pulmonary arterial pressure (prv and pap), and (c) left and right ventricular volume (Vlv and Vrv) signal waveforms over a cardiac cycle. Figure S2: (a) Internal carotid, vertebral and basilar arterial flow rate (Qica, Qva, and Qba) signal waveforms over a cardiac cycle, (b) anterior, middle, and posterior cerebral arterial flow rate (Qaca, Qmca, and Qpca) signal waveforms over a cardiac cycle. [file CNS-32-e70821-s001.docx]

**Supplementary Material**

Abbreviations used in Figure 1 are given in Supplementary Table 1, and the set values for the resistance (R), inertance (L) and compliance (C) in the cerebrovascular compartments are given in Supplementary Table 2.

**Supplementary Table 1.** Glossary of abbreviations.

| Nomenclature | |  |  |  |
| --- | --- | --- | --- | --- |
| p | Pressure |  | vp | Pulmonary veins |
| R | Resistance |  | lica | Left internal carotid artery |
| L | Inertance |  | lva | Left vertebral artery |
| C | Compliance |  | lpca | Left posterior cerebral artery |
| c | Content |  | lpcoa | Left posterior communicating artery |
| MV | Mitral valve |  | lmca | Left middle cerebral artery |
| AV | Aortic valve |  | lsca | Left superior cerebellar artery |
| PV | Pulmonary valve |  | loa | Left ophthalmic artery |
| TV | Tricuspid valve |  | laca | Left anterior cerebral artery |
| CBF | Cerebral blood flow |  | lacha | Right anterior cerebral artery |
| MAP | Mean arterial pressure |  | ba | Basilar artery |
| Subscripts |  |  | acoa | Anterior communicating artery |
| aCO2 | Arterial carbon dioxide |  | rica | Right internal carotid artery |
| aO2 | Arterial oxygen |  | rva | Right vertebral artery |
| la | Left atrium |  | rpca | Right posterior cerebral artery |
| lv | Left ventricle |  | rpcoa | Right posterior communicating artery |
| ao | Aorta |  | rmca | Right middle cerebral artery |
| aa | Aortic arch |  | rsca | Right superior cerebellar artery |
| ars | Systemic arterioles |  | roa | Right ophthalmic artery |
| cs | Systemic capillaries |  | raca | Right anterior cerebral artery |
| vs | Systemic veins |  | racha | Right anterior cerebral artery |
| ra | Right atrium |  | pc | Pial circulation |
| rv | Right ventricle |  | cc | Cerebral capillaries |
| ap | Pulmonary artery |  | vc | Cerebral veins |
| lap | Left pulmonary artery |  | l | Left |
| rap | Right pulmonary artery |  | r | Right |
| arp | Peripheral arterioles |  | 1, 2 | Segment 1 or 2 |

**Supplementary Table 2.** Set values for the resistance (R), inertance (L) and compliance (C) in the cerebrovascular compartments, left and right compartments assumed to have the same R, L and C properties, therefore, the same parameter values were used in the simulations.

|  | R [mmHg/mLs] | L [mmHg/mLs^2^] | C [mmHg/mL] |
| --- | --- | --- | --- |
| ICA | 4 | 1e-4 | - |
| VA | 1.7 | 1e-4 | - |
| BA | 6.5 | - | 0.001 |
| PCA1 | 4 | - | 0.001 |
| PCA2 | 4 | - | 0.001 |
| PCOA | 321.4 | - | - |
| MCA | 5 | - | 0.001 |
| SCA | 45.1 | - | - |
| OA | 125 | - | - |
| ACA1 | 4.7 | - | - |
| ACA2 | 3.2 | - | 0.001 |
| ACHA | 38.9 | - | - |
| ACOA | 53.6 | - | - |
| PC | 3.6 | - | - |
| CC | 1.5 | - | - |
| VC | 0.1 | - | - |
| L/R | 0.018 | - | - |

Left ventricular and aortic pressure (p_lv_, _pao_), right ventricular and pulmonary arterial pressure (p_rv_, p_ap_) and left and right ventricular volume (V_lv_, V_rv_) signal waveforms over a cardiac cycle are given in Supplementary Figure 1 and internal carotid, vertebral and basilar arterial flow rate (Q_ica_, Q_va_, Q_ba_) signal waveforms over a cardiac cycle, anterior, middle and posterior cerebral arterial flow rate (Q_aca_, Q_mca_, Q_pca_) signal waveforms over a cardiac cycle are given in Supplementary Figure 2.


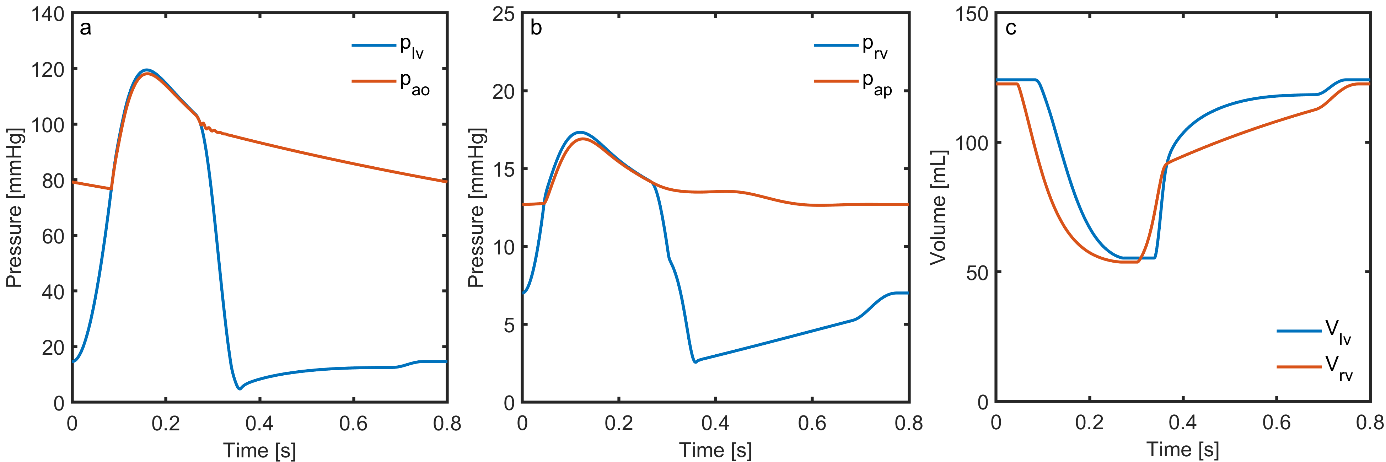


**Supplementary Figure 1.** (a) Left ventricular and aortic pressure (p_lv_, _pao_), (b) right ventricular and pulmonary arterial pressure (p_rv_, p_ap_) and (c) left and right ventricular volume (V_lv_, V_rv_) signal waveforms over a cardiac cycle.


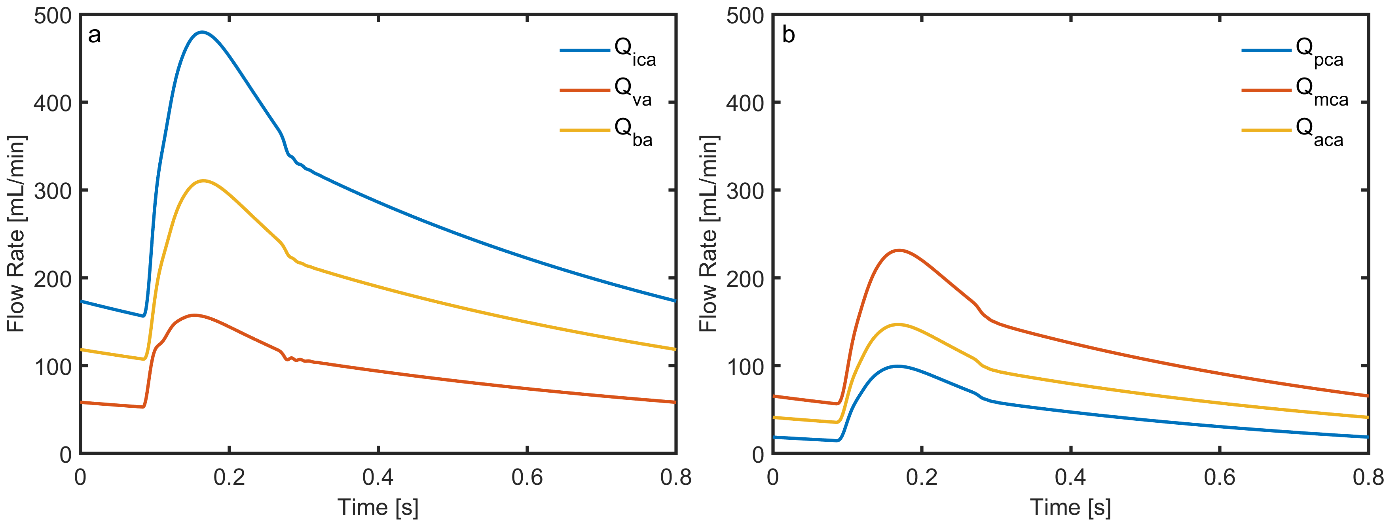


**Supplementary Figure 2.** (a) Internal carotid, vertebral and basilar arterial flow rate (Q_ica_, Q_va_, Q_ba_) signal waveforms over a cardiac cycle, (b) anterior, middle and posterior cerebral arterial flow rate (Q_aca_, Q_mca_, Q_pca_) signal waveforms over a cardiac cycle.

Aortic pressure, cardiac output, total cerebral blood flow and blood flow rates through internal carotid arteries (ICA), vertebral arteries (VA), basilar artery (BA), anterior cerebral arteries (ACA), middle cerebral arteries (MCA) and posterior cerebral arteries (PCA) over a cardiac cycle at set values for the evaluated variables in the sensitivity analysis are given in Supplementary Table 3.

**Supplementary Table 3** – Aortic pressure, cardiac output, total cerebral blood flow and blood flow rates through internal carotid arteries (ICA), vertebral arteries (VA), basilar artery (BA), anterior cerebral arteries (ACA), middle cerebral arteries (MCA) and posterior cerebral arteries (PCA) over a cardiac cycle at set values for the evaluated variables in the sensitivity analysis.

|  | Average | Diastolic | Systolic |
| --- | --- | --- | --- |
| Aortic Pressure [mmHg] | 92.1 | 76.7 | 118.2 |
| Pulmonary Arterial pressure [mmHg] | 13.7 | 12.7 | 16.9 |
| Cardiac Output [L/min] | 5.2 | - | - |
| Cerebral Blood Flow [mL/min] | 732 | - | - |
| ICA Flow [mL/min] | 275 | 157 | 480 |
| VA Flow [mL/min] | 92 | 53 | 157 |
| BA Flow [mL/min] | 182 | 107 | 310 |
| ACA Flow [mL/min] | 76 | 36 | 147 |
| MCA Flow [mL/min] | 119 | 57 | 231 |
| PCA Flow [mL/min] | 45 | 15 | 99 |
